# The study of social animal migrations: a synthesis of the past and guidelines for future research

**DOI:** 10.1098/rspb.2024.2726

**Published:** 2025-06-18

**Authors:** Iris Daniëlle Bontekoe, Ellen O. Aikens, Anna Schlicksupp, Lara Blumenstiel, Ricardo Gamito Honorato, Isabel Jorzik, Andrea Flack

**Affiliations:** ^1^ Collective Migration Group, Max Planck Institute of Animal Behavior, Konstanz 78467, Germany; ^2^ Department of Migration, Max Planck Institute of Animal Behavior, Radolfzell 78315, Germany; ^3^ Department of Biology, University of Konstanz, Konstanz 78457, Germany; ^4^ Centre for the Advanced Study of Collective Behaviour, University of Konstanz, Konstanz 78468, Germany; ^5^ School of Computing, University of Wyoming, Laramie, WY 82070, USA; ^6^ Haub School of Environment and Natural Resources, University of Wyoming, Laramie, WY 82072, USA; ^7^ Faculty of Medical and Life Sciences, Furtwangen University, Villingen-Schwenningen 78054, Germany; ^8^ Department of Chemistry, University of Konstanz, Konstanz 78457, Germany

**Keywords:** seasonal animal migration, social influences, migration decisions, systematic literature review

## Abstract

Seasonal migration is a common behaviour seen in many species worldwide. There is evidence that social factors influence various migration decisions, but compared to the well-studied field of social foraging, the study of social animal migration is still underdeveloped. Nevertheless, a few innovative studies have shown the impact of social influences on migrating animals using different tools and techniques. In this review, we systematically examined the literature to identify what makes a strong study in the field of social animal migration. We synthesized existing literature to provide advice that can improve study design and the conclusions drawn for future studies on social migration. Our analysis revealed that social interactions are widely studied among migrating species. We identified methods and approaches that, when combined correctly, provide strong evidence of social influences during migration. Finally, based on the strengths and shortcomings of past studies, we propose a set of guidelines to create more robust studies on social animal migrations.

## 1. Introduction

Seasonal migration is a widespread phenomenon occurring in many taxa across the globe. Migration involves travelling through unfamiliar or challenging conditions, thereby affecting migrants’ mortality risks [1,2] and reproductive success [3]. Considering these important fitness implications, it is likely that migrants rely on social influences during migration to improve their decision-making [4]. Social influences encompass the use of social information (e.g. local enhancement [5,6]), social learning (where information passes from informed to naïve individuals [7]) and migratory culture (migration behaviour that is socially learned, shared within a group and persistent over time [8,9]). We know that social influences can affect the various components of migration behaviour, specifically decisions regarding migration propensity, routes, timing and costs [4,10,11]. However, research on social animal migrations remains underdeveloped, especially compared to the field of social learning and culture during foraging behaviour, where the empirical support has advanced strongly over the past decades [9,12]. This difference may be because migration often occurs over large geographical areas, making it logistically more challenging to observe behaviour in detail or to perform experiments at suitable spatial and temporal scales. It is also difficult to simultaneously monitor entire groups of migrating animals, even during a small part of their journey. Similarly, the sensory modalities through which social information is transmitted are often poorly understood [13–15], hindering comparisons of the effects of social influences across different species. These challenges may even be amplified by differences between fields when inferring social information use, social learning or culture (e.g. collective behaviour versus movement ecology). For example, movement ecology examines animals in the wild, often using tracking technologies and focuses, among other things, on the social and asocial drivers of movements. The field of collective behaviour historically grew from quantifying the influence of social interactions during decision-making using controlled laboratory-based studies and theoretical models, and only in recent years, it has moved into the field [16].

Despite these difficulties, some innovative studies have succeeded in revealing the effects of social influences on migrating animals using various tools and techniques. Long-term observational studies have collected convincing evidence on social learning of migration routes or on migratory culture by monitoring multiple generations or by using a diverse set of methods [17–19]. For example, natal dispersal distances of light-bellied Brent geese (*Branta bernicla hrota*) were inferred from a combination of pedigree and observational data to reveal the role of cultural information in the development of migration routes and migratory connectivity [17]. Given the wide range of approaches used to provide evidence of social factors shaping migration, the questions arise: what types of migratory decisions are influenced by social processes? What approaches are used to study different social processes or migration decisions, and what gaps exist in the literature? Finally, what constitutes a robust study that is advancing the field of social animal migration?

In this review, we provide a quantitative overview of past research in the field of social animal migrations, complementing existing papers that review the role of social behaviour during migration [4,10,20]. In addition, we develop guidelines and examples that improve study designs and robustness, to enable researchers to reveal the occurrence of and mechanisms underlying social influences on animal migration. Therefore, we performed a systematic literature review to identify and evaluate studies that link social processes with migration decisions. We first extracted the taxonomic group of the study species, as well as the study location and the methodological approaches. Second, we identified and categorized the examined social processes and migration decisions to uncover potential biases. Third, we assessed the strength of the evidence of these studies, using predefined criteria that estimated the robustness of the scientific methods. Finally, we build upon this extracted information to develop guidelines that improve the strength of evidence regarding social animal migrations.

## 2. Details of systematic literature review

We systematically reviewed the scientific literature that was published before 1 January 2024. To do this, we performed a Clarivate Web of Science search using identified keywords describing social influences and migration. To reduce the number of non-relevant papers, we excluded some Web of Science Categories, as well as some keywords (see the electronic supplementary material for the full search string). The Web of Science search resulted in 2727 papers that we evaluated against our criteria. We only included the document types ‘Article’, ‘Article; Early Access’ and ‘Article; Proceedings Paper’ to exclude communications, such as conference abstracts and short notes. Literature reviews were excluded; however, meta-analyses were included as these provide new data-based insights. We included studies on seasonal animal migration, which we defined as movement that is predictable in both space and time and repeated by the same population on an annual basis. This excluded, for example, diel vertical migration and nomadism. We also excluded papers that were not written in English.

Relevant studies investigated at least one social process and one migration decision. We classified social processes as: (i) social information use, (ii) social learning, and (iii) culture (see the electronic supplementary material for definitions). The category of social information use included processes like collective behaviour, collective sensing, following behaviour and the presence of social information. We categorized migration decisions into four groups: (i) where-decisions, including choices on routes, stopover sites and seasonal ranges; (ii) when-decisions about the timing of migration events, i.e. departure and arrival times; (iii) whether-decisions covering migration propensity (migrant versus resident); and (iv) why-decisions, which include consequences for survival, food intake and energy expenditure. These categories were not mutually exclusive, as studies could be assigned to multiple social and migration categories. We only included studies that investigated how the social process(es) influence(s) the migration decision(s). We excluded cases where social interactions occurred across trophic levels (e.g. predator–prey and parasite–host interactions) and competitive interactions. Studies performed within a stopover site were excluded because they mainly focus on fine-scale foraging behaviour rather than on migration. We screened papers in three steps: first by the title, then by the abstract and finally, by the full content. In each phase, we excluded papers that did not meet our selection criteria.

Our final selection included 116 papers. From these papers, we extracted the following information: *type of study* (empirical, theoretical or a combination of both), *taxonomic group* of the study species and *geographical location* of the study (continent and location where the study was performed). Studies could be assigned to more than one continent if data from multiple continents were used. For biologging studies, we used the location of tag deployment as the study location. We also extracted the *location of the first author’s primary affiliation* and determined which *social processes* and *migration decisions* were investigated. In addition, we estimated the *spatial and temporal scales* at which the social processes and migration decisions were studied (see the electronic supplementary material for definitions). We determined the *methods* used (e.g. remote individual tracking, counts and theoretical modelling; see the electronic supplementary material for a full list and details) and classified the *study approach*, which included categories for observations of natural behaviour, reintroductions, manipulations and others (see the electronic supplementary material for definitions).

Finally, for each empirical study, we assessed the robustness of the overall scientific approach on different axes. We specified that a study delivers ‘strong evidence’ when it develops and excludes alternative hypotheses, performs well-designed experiments [21] and/or directly measures social influences on migration decisions. Therefore, our axes to assess the strength of evidence include *hypothesis testing, an inferential approach* and *quantification of social influence*. The axis of *hypotheses testing* has the following categories ranked from lowest to highest scientific robustness: (i) no hypotheses, (ii) vague hypotheses that are not clearly stated and without clear predictions, (iii) clearly stated hypotheses that are not mutually exclusive, and (iv) multiple, mutually exclusive hypotheses. The *inferential approach* was categorized (lowest to highest) as: (i) observational, (ii) suboptimal laboratory experiment, (iii) suboptimal field experiment, (iv) optimal laboratory experiment, and (v) optimal field experiment (definitions in the electronic supplementary materiall). Finally, we quantified the *degree to which social influences were quantified* to define the strength of the observed relationship between social processes and migration decisions. The *quantification of social influence* was ranked (lowest to highest) as follows: (i) social influence discussed (i.e. no direct measurements of social factors), (ii) social influence inferred (i.e. the conditions for a link between social processes and migration decisions are shown but no direct measurements are made; e.g. co-occurrence between adults and juveniles), and (iii) social influence measured (i.e. the direct effect of social processes on migration decisions was quantified; e.g. the measured response of individuals to an artificial social cue).

Each paper was reviewed by at least two people (out of the eight people responsible for reading and reviewing studies) to increase the consistency in data collection.

## 3. Synthesis of existing studies

**Figure 1.**
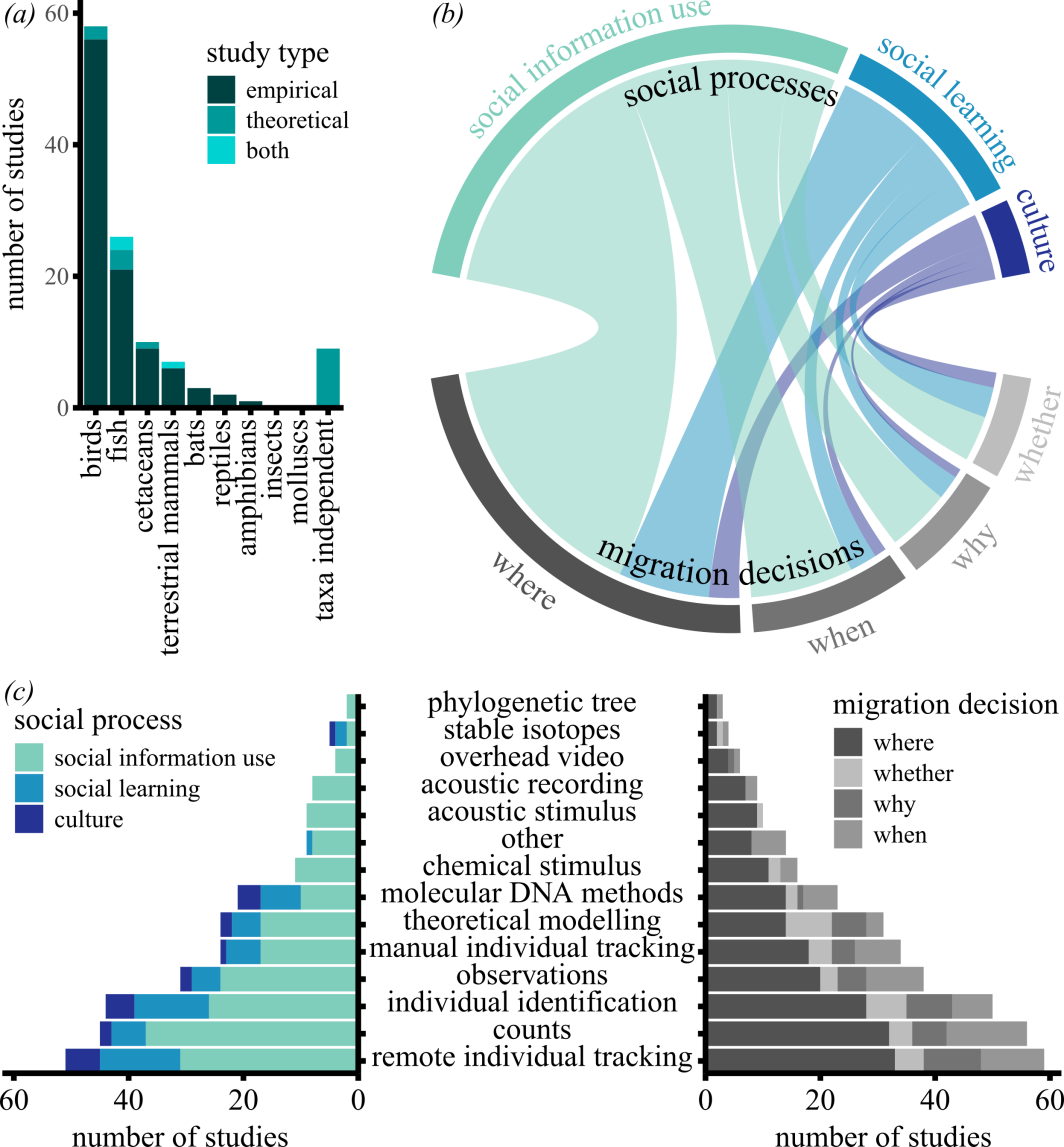
An overview of the taxa and of the social processes and migration decisions that have been studied in the past, as well as the methods that were used. (*a*) The number of studies per taxonomic group, and the colours indicate the study type. (*b*) Connections between social processes and migration decisions, with the width of connections being indicative of the number of studies. (*c*) The number of studies that used a certain method (middle) to study social processes (left) and migration decisions (right), with colours corresponding to the different categories.

Studies on social migration encompassed a range of taxa, spatiotemporal scales and methodological approaches. Yet, there were taxonomic and geographical biases as most studies focused on birds (50%; figure 1*a*), followed by fishes (22%), cetaceans (9%), terrestrial mammals (6%), bats (3%), reptiles (2%) and amphibians (1%). Only 8% of the studies were taxa-independent. These biases are comparable to those in the fields of ecology, zoology and behaviour, where the number of species in each taxonomic group does not correspond with the number of published papers [22,23]. Birds are also often over-represented in biodiversity research [24], and even within taxonomic groups, there are often biases, with some species being studied more than others [25]. Despite the global nature of animal migrations, studies from North America (47%) and Europe (42%) were over-represented in our selection. This pattern of geographical bias was consistent with the first author’s primary affiliation (see the electronic supplementary material, figure S1). Our finding matches the geographical bias in the overall published literature [26,27] and may have been amplified by our use of English search terms and the exclusion of non-English publications [28].

We also uncovered biases in the social processes and migration decisions that were studied. Most studies assessed the effects of social information use (88%; figure 1*b*), whereas only 11% of the studies investigated migratory culture. This is probably because social information use occurs at a relatively short spatial and temporal scale (e.g. [29]), which makes it easier to design and perform studies on social information use. While it is possible to investigate social learning and culture at a fine spatial and temporal scale (e.g. [19,30]), studying culture often requires decades of data collection to make conclusive statements (e.g. [31]). The presence of studies on migratory culture in our selection highlights that it is possible to study culture at different spatial and temporal scales. We found a general match between the timescale of the social processes and migration decisions being studied (electronic supplementary material, figure S2). Individual-based movement studies paired with data on social relations (e.g. pedigree data) can provide a more detailed understanding of the impact of social interactions on migration [32].

Most studies investigating social influences focused on where-decisions (85%; figure 1*b*), with when- (31%), why- (20%) and whether- (16%) decisions being less frequent. Different methods, ranging from biologging to DNA sampling (figure 1*c*), were often used to study spatial migratory decisions (i.e. where-decisions). However, despite temporal decisions having strong fitness implications for migrants [33], the effect of social influences on when-decisions was studied less frequently. Studying why-decisions (i.e. looking at costs and benefits) requires information on energetic costs, long-term reproductive success and/or survival. These factors are often challenging to obtain but highly relevant for both conservation purposes (e.g. [34,35]) and for contextualizing the role of social migration in an eco-evolutionary framework. Finally, the impact of conspecifics on the fundamental decision of whether to migrate or not is also an understudied subject, most likely because it requires monitoring a large proportion of the population. Nearly half of the studies on whether-decisions (42%) were theoretical studies, highlighting the benefits arising from theoretical studies in a field where it is genuinely hard to empirically quantify individual decisions and their dependence on social interactions. Theoretical modelling is commonly used to gain insights by simulating complex dynamic processes like density dependence, collective effects and anthropogenic impacts [36–38]. These studies also show that we have already begun to establish a theoretical foundation that is now ripe for expansion and empirical investigation.

The majority of studies were empirical (84%), while 13% of the studies were theoretical and only 3% contained both an empirical and a theoretical component. Of the empirical studies, 82% observed natural behaviour, whereas a small number of studies used experimental manipulations (28%) or reintroductions (8%). We identified a wide variety of methods to investigate how social influences affect migration decisions. Across all taxa, counts (36%) were used most, followed by remote individual tracking (29%), individual identification (28%) and direct visual observations (22%). This large variation in methods may be driven by variations in body size, social organization, habitat, communication style or lifespan of the focal taxa. Generally, we found that social migrations of larger species (e.g. large bird species, mammals or reptiles) were often examined using remote individual tracking, such as GPS or satellite tracking [39–43]. Using these technologies, several studies examined where-decisions, including collective movements [44–46], or the cultural transmission of routes and timing [31,47]. Social ties of smaller species, like small bats, birds and most fish, were often explored using counts, individual identification, or molecular DNA methods [48–51], determining whether related individuals co-occurred at the same time and place (where-decisions; [48,52]). A few studies also combined these methods with artificial stimulation using odour or sound to explore the effect of social information use on the timing of migration (when-decisions; [53,54]). Marine mammals were often studied using individual identification and molecular DNA methods to identify family ties of groups and provide insights into social learning and culture [55]. Several studies on bats, cetaceans and birds have used stable isotope analyses to approximate the locations of routes and seasonal ranges of social groups (where-decisions; [48,56,57]).

## 4. Strength of empirical evidence

Next, to go beyond an overview of the biases in existing literature, we estimated the strength of evidence for each study (based on our definition; see §2). By evaluating the scientific robustness of the applied overall study approach, we identified potential shortcomings in existing studies. We then used this information to provide guidelines for future research, with the aim to increase the robustness of studies on social animal migrations and to obtain stronger evidence.

**Figure 2.**
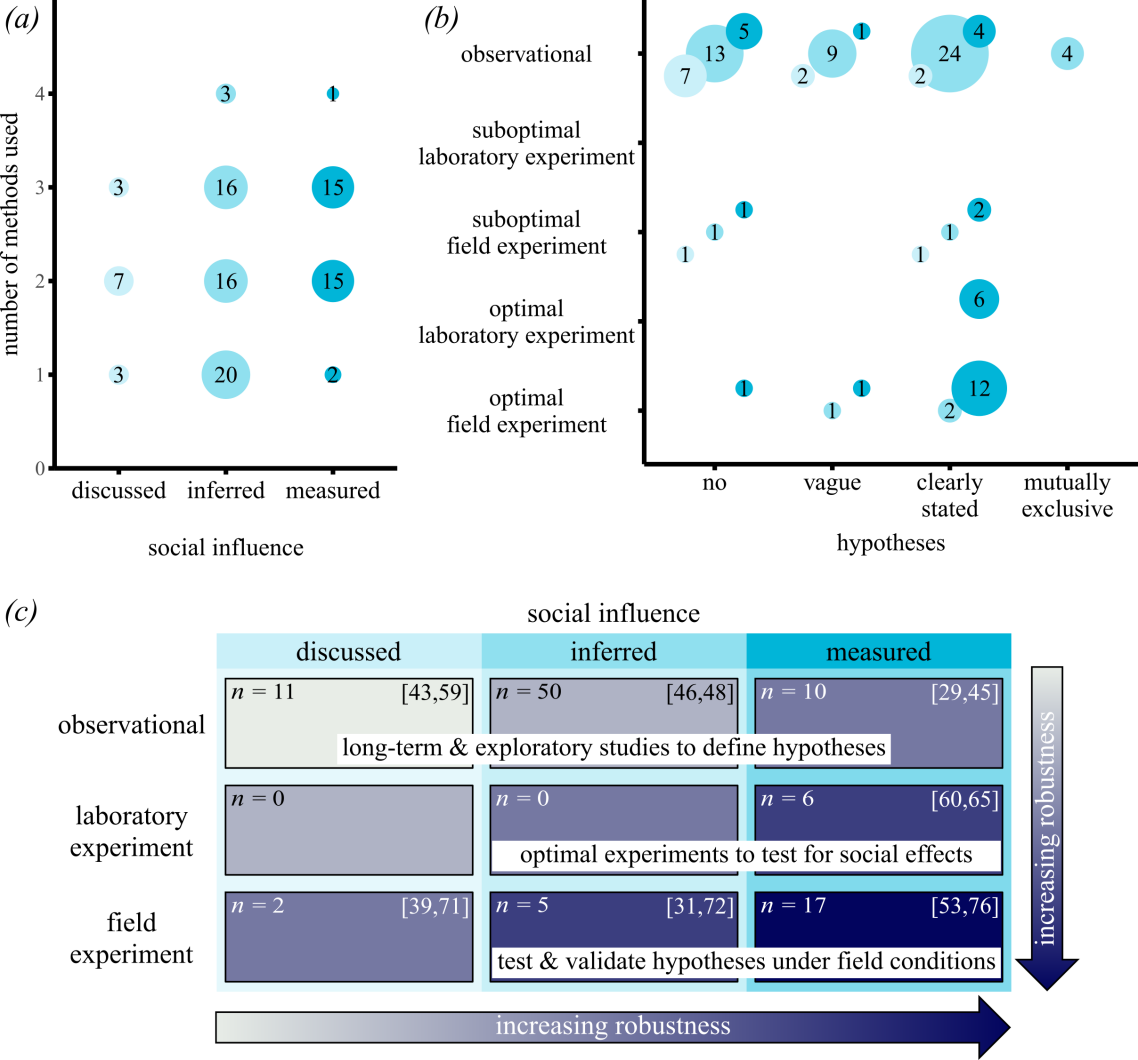
Quantification of studies in terms of the number of methods used and the different axes of scientific robustness. (*a*) Relationship between the quantification of social influence and the number of methods used. The circle area and numbers inside the circles represent the number of studies that fall into the respective categories. Colours represent the categorization of the quantification of social influence. (*b*) Relationship between hypothesis testing, the inferential approach and quantification of social influence. The circle area and numbers inside the circles represent the number of studies that fall into the respective categories. Colours represent the categorization of the quantification of social influence. (*c*) Case studies identified via a systematic review were placed within our classification of scientific robustness (i.e. quantification of social influence versus inferential approach). Sample sizes within each box represent a number of studies that fall into the respective categories.

Based on our estimation of scientific robustness, we scored each empirical study on the following axes: hypothesis testing, quantification of sociality and the inferential approach (figure 2). None of the studies fell into the highest category on all axes (i.e. multiple, mutually exclusive hypotheses were evaluated, sociality was directly measured and field experiments with strong controls and replication were performed; see §2). Strikingly, we identified very few studies (4%) that defined and tested mutually exclusive hypotheses (e.g. [58]). In fact, over one-third of the studies either did not state clear hypotheses (14%) or stated no hypotheses (29%) regarding the effect of social influences on migration. However, note that when the studies’ hypotheses covered aspects other than social influences on migration (e.g. [59]), we classified this as ‘no hypotheses’. As stated before, the large majority of studies were observational rather than experimental (figure 2*b*), with most experiments (80%) performed in the field (mainly on birds and fishes) and fewer in artificial settings (i.e. in the laboratory, mainly on fishes, birds and amphibians). The majority (71%) of the field experiments had control groups and/or replicates, while this was the case for all laboratory experiments (figure 2*b*). One-third of the studies measured social influences empirically (e.g. by quantifying leader–follower interactions), while more than half (54%) of the studies only inferred how social influences affect migration behaviour. The majority (70%) of the studies that measured social influences on migration were experimental, measuring, for example, the effects of social influences by presenting a specific social stimulus (e.g. [60–65]). The majority of empirical studies combined multiple methods to investigate social influences on migration (figure 2*a*).

Although only a minority of the studies reached high scores in our classification categories, it is essential to also carefully examine those studies that did well in one of the categories. For example, a study on American black bear (*Ursus americanus*) migration [58] relied on a single method (i.e. radio tracking), but the authors defined multiple hypotheses and predictions based on the literature to then compare those with their observational data. By carefully testing ecological theories, observational data can lead to new insights into the field of social animal migrations that can then be further examined in novel, targeted experiments. In addition, bio-logging devices are particularly well-suited for such ‘single method’ studies to test and refine ecological theory, as they allow researchers to apply a ‘big-data’ approach with adequate sample sizes and enable in-depth analyses [66,67]. However, when designing new studies to explore how social processes influence migration decisions, strong scientific evidence can also be achieved by combining approaches (e.g. manipulations and observing natural behaviour) and complementary methods (e.g. remote individual tracking, observations and molecular DNA methods). For example, studies on whooping cranes (*Grus americana*) have used tracking technologies in combination with an experimental approach and data from resightings of individuals and groups. This allowed the authors to relate detailed observations on group size and composition (i.e. the quantified social environment) to the fine-scale movement tracks [68,69]. In addition, the study species was part of a reintroduced population, where each individual is known and monitored. This enables researchers to study the movements and social interactions of the entire population, while controlling for the potentially confounding effects of genetics. In general, reintroduction programmes provide a good opportunity to study social migration, as these often provide long-term pedigree data, whole-population tracking and real-time monitoring of individuals [31,70].

Thus, experiments conducted in the wild can result in strong evidence. However, it is challenging to create similar levels of robustness as laboratory experiments, because it is not always possible to reach large sample sizes [71] or to develop the strongest controls [72] in natural settings. By contrast, experiments in the wild have the advantage of being conducted in a natural physical and social environmental context [73]. Behavioural responses to social stimuli may be different in laboratory settings than in the wild [74]. In addition, the response to social information from conspecifics may be different depending on the experiences and states of the interacting individuals [47,75]. The trade-off between the experimental principles of replication, randomization and strong controls on the one hand and environmental context on the other hand may result in oversimplified experimental studies. However, even simplified studies can lead to new insights into the understudied topic of social animal migrations. For example, simple experiments, such as presenting acoustic [60,63,76], chemical [77,78] or visual stimuli [5] in the laboratory or field, can indicate whether individuals are attracted to (or repelled by) the presence of others. Further, performing, for example, playbacks in unsuitable habitats, increases the strength of evidence, as an individual’s response is probably owing to the social stimulus [79].

## 5. A call for more robust studies on social animal migrations

Together, this synthesis highlights that more work is needed to provide robust evidence of the influence of various social processes on various migration decisions. Future studies should focus on developing and testing mutually exclusive hypotheses, performing creative experiments in natural settings and directly quantifying the social processes shaping migratory decision-making. Inspired by the strengths of past studies, we provide a set of guidelines to generate more robust studies on social animal migrations. The following guidelines are not exhaustive, but instead are intended to inspire an ongoing conversation about future research on social influences during migration. Finally, in box 1, we provide three open example questions originating from three recent reviews on social migrations [4,10,20] and provide recommendations on how these could be answered, considering the following guidelines.

Box 1. **Suggestions on how to answer open questions in social animal migration research.**

**Do migratory insects benefit from collective navigation?** From Berdahl *et al.* [20]

Using an interdisciplinary approach, it will probably soon be possible to examine whether, and if so, how social processes influence insect migrations. The larger insect species can already be tracked with biologging devices [80], and vertical-looking radars have allowed long-term monitoring of the altitudinal and temporal dynamics of high-flying insect populations [81], a first step to understand potential collective effects [82]. In recent years, virtual reality systems have been used to study insect migrations experimentally to test how, for example, landmarks (e.g. [83]) or the sun (e.g. [84,85]) affect migratory decisions. These studies could be extended to test how migrating insects (and later larger species) respond to virtual conspecifics at short spatial and temporal scales. Such virtual reality experiments, including experiments on social influences, have been performed on small laboratory animals [86,87].

**How do migratory animals gain information from other species?** From Aikens *et al.* [4]

A series of field and laboratory-based experiments using different spatial and temporal scales could examine whether species respond to and gain information from heterospecifics. First, attraction or avoidance of other species could be examined by recording individual responses to acoustic, chemical, or visual stimuli. These types of experiments can be performed on relatively small spatial and temporal scales and focus on how social information use influences where-decisions. To extend these experimental approaches to other migration decisions, researchers could, for example, present acoustic stimuli at a stopover site for migratory birds under a range of favourable and unfavourable migration conditions to understand the effects of social cues on when-decisions. More elaborate experiments could involve translocations of wild animals.

**How can we more effectively differentiate the role of social cues from synchronous, independent responses of individuals to an environmental cue?** From Oestreich *et al.* [10]

Experimental studies on soaring birds using a combination of high-resolution tracking devices and drones could be used to estimate the collective benefits of flying in flocks. Autonomous soaring drones could be used as a source of social information to examine whether wild migrants would respond to efficiently soaring agents in the sky even when the environmental conditions and migration timing are not ideal.

### (a) Defining hypotheses and predictions

Most studies in our selection were observational studies. Insights from these short- and long-term studies should be used for generating multiple, mutually exclusive hypotheses with clear predictions [31,39], which so far still happens rarely in the field (figure 2*b*,*c*). For example, by simply monitoring the migratory behaviour of juveniles and parents (or young and old individuals), we can develop hypotheses on how strongly migratory decisions are influenced by social learning and culture [18,88]. As a next step, we can test these hypotheses in natural settings [69] or specifically designed [72] experimental settings. In addition, agent-based models could be used to shape [20] and evaluate [89] multiple hypotheses (also see below). Yet, when designing studies, it is important to consider the various temporal scales at which social processes may influence migration decisions.

### (b) Integrating multiple timescales

Social processes during migration can be studied at various scales (electronic supplementary material, figure S2). Long-term processes like migratory culture require long-term data, however, to understand the detailed behavioural mechanisms by which social information is transferred and affects migration, we need studies focusing on fine-scale processes. Social information use is a prerequisite for social learning, which, in turn, is a prerequisite for culture [4]. Therefore, studies that cover multiple timescales and assemble the various layers of social influences are needed. This means that researchers should develop methods for integrating different timescales into data collection to then investigate both short- and long-term social influences simultaneously [47,68]. For example, studies with well-designed experiments have shown that individuals respond to social information when making their migratory decisions [79,90,91]. While these studies are valuable in uncovering behavioural mechanisms, individuals are not followed long-term, and therefore, no measurements on longer-term social influences can be made. To study migration culture, researchers have used molecular DNA methods [19,30,56] or multiple years of individual occurrence data [17,92]. Using such a method, it is not possible to investigate fine-scale behavioural mechanisms. Therefore, researchers need to carefully choose and combine suitable methods to optimally investigate social influences at multiple timescales. Similarly, researchers should aim to design studies that investigate social influences on multiple migration decisions.

### (c) Adopting interdisciplinary approaches

We need to bring complementary research fields (e.g. theoretical biology and field ecology) together to reveal the impact of social influences on migrants. For example, theoretical models, such as individual/agent-based models (e.g. [93]), could test simple interaction rules that are expected to play a role in the wild. These theoretical patterns could be compared to empirical movement patterns (e.g. [94]) to gain insights into social interaction rules that shape migration. Further, individual-based models can be a valuable tool to bridge empirical observations at individual-, group- and population-level (and at different timescales) to explore social influences on migration decisions and test multiple hypotheses about migration decisions [89]. We also suggest combining studies in the laboratory and in the field [95] to gain a more holistic understanding of social influences on migration. In addition, there is a variety of fields that could shed new light on social migration research, including the fields of sensory ecology [13,15,96,97], neurobiology [98,99], computer science and engineering [100,101], genetics and molecular biology [8,17,102], evolutionary ecology [103] and/or field and laboratory-based behavioural ecology [54,91] (also see box 1 for examples).

### (d) Adopting novel tools and technologies

Carefully quantifying the effect of social influences on migration decisions can be improved by adopting and combining various methods (figures 1*c* and 2*a*). For example, using multiple methods to address questions on social migration (or more broadly social behaviour) enables distinct observation modes at multiple levels of biological organization.

Thus, we need to creatively link new and old technologies to reveal the effect of social influences on migration at the various organizational levels. For example, increasingly small and cheap biologging devices enable researchers to simultaneously track large numbers of migrating individuals [104,105], making bio-loggers ideal for individual-level (occasional group-level) observations [66]. Virtual reality experiments may soon be able to show how social processes influence navigational decisions during migration (e.g. [84,86,106,107]), and physiological measurements can directly quantify the costs of social interactions in the laboratory and the field [108,109]. Census data through drones and other imaging devices (e.g. [29,100]) provide mainly population or group-level observations by generating reliable estimates of the numbers of animals, the structure of their environment and the interaction of animals with each other and with their habitat [101,110,111]. Similarly, passive acoustic recordings and radar observations can provide information on the social context in which animals are migrating at the population level [81]. Further, acoustic signals often encode detailed information about individuals’ behavioural state, allowing the assessment of both the signalling individual’s behaviour and its social influence on the behaviour of others [112].

Thus, similar to the various timescales, integrating observations across different levels of biological organization [113] is critical for inferring the role of social influences in migration behaviour.

### (e) Considering ethics and animal welfare

Large parts of the research described or proposed here have ethical and welfare implications. Designing studies, therefore, requires careful assessment of potential impacts on the animals [114–116]. Further, when combining methods and approaches, it is important to consider the cumulative impact of these methods on animals and ecosystems [116]. For example, experimental manipulations may not only involve carrying biologging devices, but can also have other consequences (e.g. translocated animals may experience reproductive disadvantages or even death; [117]). Thus, it is essential to reduce any negative impacts to a minimum by, for example, combining empirical data with theoretical models (e.g. [44,94]; see above) to reduce the need for invasive approaches, or by, for example, using biologgers that detach after data collection [116]. Further, it is important to make stronger predictions about natural processes, enabling the development of more conclusive experiments (see above). In addition to the influence of research activities on animals, certain methods (e.g. camera trapping, acoustic recordings, collection of environmental DNA) may raise human-related ethical concerns as well when data affecting human privacy are collected as a byproduct (e.g. [112,118,119]).

Thus, when designing new studies, it is important to keep ethics and animal welfare in mind and consider the trade-off between knowledge gain and the costs borne by the animals. In the case of threatened species, researchers and conservationists should collaborate to assess whether, for example, the use of biologging devices imposes an unnecessary burden on these already vulnerable animals, despite the valuable insights that may be gained.

### (f) From research to conservation and back

Social influences probably shape the ability of many migratory animals to adjust to environmental change; therefore, understanding social learning and culture matters for conservation [4,120]. Future studies should aim at providing guidelines for conservation actions. At the same time, conservation actions, especially those involving reintroductions, translocations and captive breeding programmes, can be used to study the influence of social processes during migration (e.g. [31,68]).

## 6. Conclusions

Our systematic literature review has highlighted that social interactions are abundant across migrating taxa. We identified methods and approaches that, when used in the right combination, can provide strong scientific evidence of the impact of social influences during migration. However, we also revealed that there are still shortcomings in the existing studies, as they often do not define multiple, mutually exclusive hypotheses, or only rarely perform well-designed field experiments to test for the impact of social influences. We acknowledge the challenges associated with field experiments but also provide a set of guidelines that may improve future endeavours to study social animal migrations.

## Data Availability

The data and code used for this study are available through Edmond—the Open Research Data Repository of the Max Planck Society [121]. Electronic supplementary material and figures are provided online [122].
